# Renal cortical T1 times at 1.5 T: exploratory reference estimates from a real-world cohort with preserved renal function

**DOI:** 10.1016/j.ejro.2026.100788

**Published:** 2026-07-02

**Authors:** L. Lunzer, K. Mascherbauer, C. Kronberger, C. Dona, C. Nitsche, M. Koschutnik, M. Poledniczek, L. Schmid, A. Geppert, D. Beitzke, C. Loewe, C. Hengstenberg, A. Kammerlander

**Affiliations:** a Medical Department, Division of Cardiology, Medical University of Vienna, Austria; b Medical Department, Division of Cardiology and Intensive Care Medicine, Klinik Ottakring, Vienna, Austria; c Division of Cardiovascular and Interventional Radiology, Department of Biomedical Imaging and Image-guided Therapy, Medical University of Vienna, Vienna, Austria

**Keywords:** Cardiac magnetic resonance, cardiorenal syndrome, renal T1 times, chronic kidney disease, reference ranges

## Abstract

**Background:**

Early detection of renal tissue alterations remains challenging, as laboratory markers often remain within the normal range until advanced stages of disease. T1 mapping offers a non-invasive approach to assess tissue composition, but data on typical renal cortical T1 times in real-world clinical populations at 1.5 T are limited.

**Aim:**

To define exploratory real-world reference estimates for renal cortical T1 times and to assess their association with demographic and laboratory parameters in individuals with preserved renal function.

**Methods:**

This retrospective study included patients undergoing clinical cardiac magnetic resonance (CMR) at 1.5 T in whom the kidneys were visible on native short-axis T1 maps. T1 times were derived from three manually placed regions of interest in the upper, middle, and lower renal cortex and averaged. Associations with sex, age, hemoglobin, and hematocrit were analysed.

**Results:**

In a cohort without relevant comorbidities (n = 65), mean renal cortical T1 times were 1054 ± 53 ms (95% CI 1040–1067). Renal cortical T1 times showed no significant difference between women and men (1068 ± 34 ms vs. 1046 ± 60 ms, p = 0.21). No significant differences were observed across age groups (p = 0.34). Renal cortical T1 times were not significantly associated with hemoglobin (r = –0.10, p = 0.45) or hematocrit (r = –0.08, p = 0.54).

**Conclusion:**

Renal cortical T1 times derived from routine CMR appear relatively stable across age and sex in individuals with preserved renal function. Opportunistic renal T1 assessment from routine CMR may provide additional, non-invasive information on renal tissue characteristics without extending scan time.

## Graphical Abstract

**Figure:**
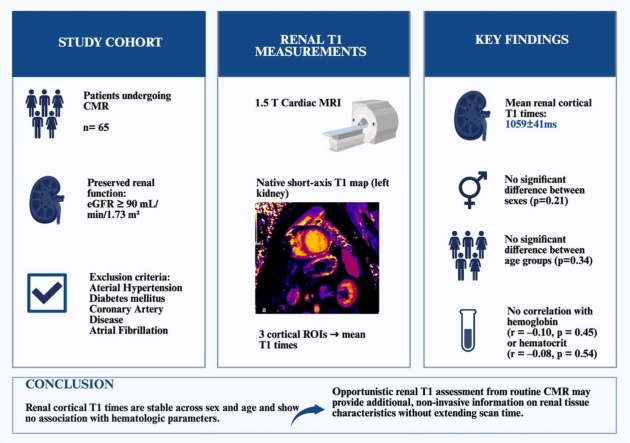
Graphical Abstract.

## 1 Introduction

Chronic kidney disease (CKD) is common in patients with cardiovascular disease and is associated with adverse outcomes. Early detection of subclinical renal involvement remains a challenge because serum markers often remain within the normal range until advanced stages of disease [1].

Magnetic resonance–based renal T1 mapping is a non-invasive technique that reflects tissue composition and may be sensitive to structural alterations, including fibrosis, edema and amyloid deposition [2], [3], [4]. Recent studies have demonstrated correlations between renal T1 times and renal function parameters, such as estimated glomerular filtration rate (eGFR) and serum creatinine. However, most previous studies have focused on small cohorts of healthy volunteers or patients with advanced CKD, limiting clinical applicability. Reference estimates, particularly at the widely used field strength of 1.5 Tesla (T), are not yet well established in real-world populations [3], [4], [5], [6], [7], [8].

To address this gap, we established renal cortical T1 real-world reference estimates at 1.5 T in a cohort with preserved renal function undergoing cardiac magnetic resonance (CMR). We also explored associations with demographic and laboratory markers.

## 2 Methods

### 2.1 Study design

This retrospective analysis was conducted within the framework of a prospective registry at the Medical University of Vienna, Department of Internal Medicine II, Division of Cardiology. The study was approved by the institutional ethics committee (EK#2036/2015) and was performed in accordance with the Declaration of Helsinki. Written informed consent was obtained from all participants prior to inclusion.

We retrospectively analysed consecutive patients who underwent 1.5 T CMR between 2013 and 2023 in whom the kidneys were visible on native short-axis T1 maps (MOLLI 5 [3]3). The cohort was defined by an eGFR ≥ 90 ml/min/1.73 m² and the absence of renal disease, diabetes mellitus (DM), arterial hypertension (aHTN), coronary artery disease, or atrial fibrillation. Regions of interest (ROIs) were placed at the superior, middle, and inferior poles of the renal cortex and averaged to obtain global renal cortical T1 times.

ROI size was adapted to the individual cortical thickness to ensure accurate placement within the cortex while minimizing partial volume effects. Due to anatomical variability and the use of cardiac short-axis images, ROI size was not strictly standardized.

While the renal medulla may be partially visible on cardiac short-axis T1 maps, its boundaries cannot be consistently and reproducibly delineated due to the oblique imaging plane, limited spatial resolution, and lack of clear corticomedullary contrast. Therefore, reliable placement of medullary ROIs was not feasible without introducing substantial partial volume effects.

Statistical analyses included descriptive statistics expressed as mean ± standard deviation (SD) with 95% confidence intervals (CIs). Sex differences were evaluated using multivariable linear regression models adjusted for age, body mass index (BMI), hemoglobin, and hematocrit.

Subjects with visible kidneys on short-axis T1-mapping sequences were included in the study. Referral indications comprised suspected valvular disease (30%), heart failure (14%), coronary artery disease (10%), storage disease (17%), myocarditis (7%), and other causes (17%).

Clinical information was obtained from medical records. Laboratory data, including serum creatinine and eGFR, were collected before CMR. eGFR was calculated using the Chronic Kidney Disease Epidemiology Collaboration (CKD-EPI) equation. [9] Participants were not required to undergo specific preparation, such as fasting. BMI was calculated as weight (kg) divided by height squared (m²).

The diagnosis of aHTN was based on office systolic blood pressure (SBP) ≥ 140 mmHg and/or diastolic blood pressure (DBP) ≥ 90 mmHg after repeated measurements [10].

DM was diagnosed according to HbA1c ≥ 6.5% (≥ 48 mmol/mol Hb) or impaired fasting glucose in the range of 100–125 mg/dL (5.6–6.9 mmol/L) in venous plasma [11].

Renal cortical T1 times were compared between age groups and sexes. To ensure a homogeneous study population and to address concerns regarding potential selection bias, only subjects with preserved renal function (eGFR ≥ 90 ml/min/1.73 m²) and without relevant comorbidities (including DM, aHTN, coronary artery disease, and atrial fibrillation) were included in the final control cohort. Further exclusion criteria included poor image quality, and lack of suitable T1 mapping.

### 2.2 Cardiac magnetic resonance imaging

All CMR examinations were performed on a 1.5 T scanner (Avanto FIT; Siemens Medical Solutions, Erlangen, Germany) according to standard protocols [12] [13]. Steady-state free-precession sequences were used for cine imaging (repetition time/echo time, 3.2/1.2 ms; flip angle, 64°; voxel size, 1.4 × 1.4 × 6 mm; matrix, 180 × 256). Late gadolinium enhancement imaging was performed using segmented inversion recovery sequences (700/1.22 ms; flip angle, 50°; voxel size, 1.4 × 1.4 × 8 mm; matrix, 146 × 256) at least 10 min after intravenous administration of 0.1 mmol/kg gadobutrol (Gadovist; Bayer Vital GmbH, Leverkusen, Germany).

T1 mapping was performed using electrocardiographically triggered MOLLI with a 5 [3]3 prototype scheme (five acquisition heartbeats, three recovery heartbeats, and three acquisition heartbeats) in short-axis and four-chamber views. Sequence parameters included initial inversion time (TI) 120 ms, TI increment 80 ms, reconstructed matrix 256 × 218, measured matrix 256 × 144 (phase-encoding resolution 66%, field of view 85%). Native (pre-contrast) T1 maps and post-contrast T1 maps **(**15 min after contrast**)** were acquired. In addition to standard cardiac T1 maps, ROIs encompassing the renal cortex were manually defined. Renal cortical T1 times were derived exclusively from native T1 maps; post-contrast maps were acquired for the cardiac protocol but were not used for renal analyses.

No dedicated protocol adjustments or preparatory measures were applied for renal imaging. All data were obtained from routine clinical CMR examinations.

### 2.3 Definition of renal T1 times

**Fig. 1:**
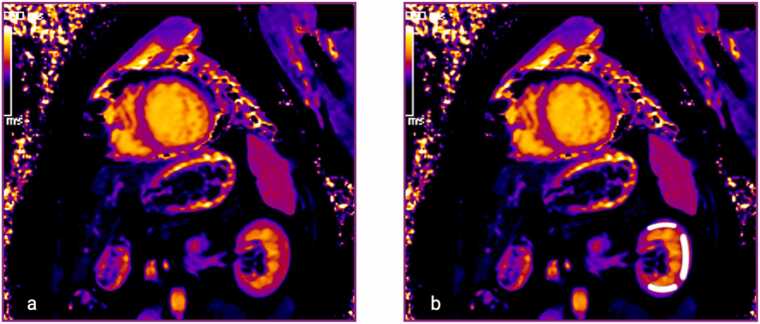
Example of a cardiac magnetic resonance (CMR) short-axis view showing the left kidney on native T1 maps (a, b). Three regions of interest (ROIs) were manually placed in the renal cortex, and corresponding T1 times were recorded in milliseconds (ms).

Renal cortical T1 times were measured on native short-axis T1 maps using a dedicated workstation (IMPAX EE R20 XV, Agfa Healthcare) (Fig. 1). To improve representativeness, three ROIs were placed in the upper, middle, and lower renal cortex and averaged to obtain global renal cortical T1 times. However, focal heterogeneity of renal tissue cannot be fully captured by this approach. Renal cortical T1 times were expressed in milliseconds (ms). Reproducibility was assessed using the intraclass correlation coefficient (ICC) based on a two-way random effects model with absolute agreement.

### 2.4 Outcome measures

The aim of this study was to define real-world reference estimates for renal cortical T1 times in patients undergoing 1.5 T CMR to support the broader clinical use of T1 mapping sequences and to provide further evidence for risk stratification in patients with renal disease.

### 2.5 Statistical analysis

Continuous variables are reported as mean ± SD or median (IQR), and categorical variables as counts (%). Normality was assessed using Kolmogorov–Smirnov tests and visual inspection of Q–Q plots. Between-group comparisons used independent-samples *t*-tests or one-way ANOVA for continuous variables and χ² tests for categorical variables. Univariable linear regression assessed associations with age, and sex-related differences were evaluated using multivariable linear regression adjusted for age, BMI, hemoglobin, and hematocrit. Two-sided p ≤ 0.05 was considered statistically significant (SPSS version 29).

## 3 Results

Baseline characteristics stratified by sex are presented in Table 1, Table 2. The cohort consisted of 65 subjects aged 18–80 years. Women had lower body mass index, hemoglobin, and hematocrit levels compared with men, while other baseline characteristics were comparable.

**Table 1:** Baseline characteristics in subjects without known comorbidities stratified by sex.

**Variables**	**Control** **(n = 65)**	**Male** **(n = 41)**	**Female** **(n = 24)**	**p-value**
	**Mean ± SD** **(95%-CI)**	**Mean ± SD (95%-CI)**	**Mean ± SD** **(95%-CI)**	
***Clinical parameters***				
Age, years	42 ± 17	41 ± 16	44 ± 19	0.56
BMI, kg/m^2^	24 ± 3	25 ± 3	23 ± 3	**0.001**
***Laboratory markers***				
Creatinine, mg/dL	0.8 ± 0.2	0.8 ± 0.1	0.6 ± 0.9	**< .001**
eGFR, ml/min/1.73m^2^	114 ± 25	112 ± 27	118 ± 23	0.41
Hemoglobin, g/dL	14 ± 2	15 ± 2	13 ± 1	**< .001**
Hematocrit, %	41 ± 4	43 ± 3	38 ± 4	**< .001**
***Cardiac magnetic resonance imaging parameters***				
LV-EF, %	61 ± 9	61 ± 9	61 ± 9	0.99
RV-EF, %	55 ± 9	55 ± 9	54 ± 8	0.67
IVS, mm	10 ± 4	11 ± 4	10 ± 3	0.13
LVEDVi, ml/m^2^	84 ± 18	89 ± 17	75 ± 17	**0.004**
RVEDVI, ml/m2	87 ± 22	94 ± 24	77 ± 13	**0.002**
Myocardial T1 times, ms	1006 ± 45	999 ± 45	1017 ± 45	0.13
Renal T1 times, global, ms	1054 ± 53(1040–1067)	1046 ± 60 (1027–1064)	1068 ± 34 (1053–1082)	0.21
Renal T1 times, upper, ms	1069 ± 58 (1054–1083)	1065 ± 66 (1044–1086)	1075 ± 44 (1056–1093)	0.13
Renal T1 times, middle, ms	1061 ± 55 (1047–1074)	1052 ± 54 (1034–1069)	1076 ± 55 (1053–1100)	0.65
Renal T1 times, lower, ms	1044 ± 58 (1030–1058)	1041 ± 58 (1022–1059)	1051 ± 60 (1025–1077)	0.83

**Abbreviations.** BMI, body mass index; eGFR, estimated glomerular filtration rate; LV-EF, left ventricular ejection fraction; RV-EF, right ventricular ejection fraction; IVS, intraventricular septum; LVEDVi left ventricular end-diastolic volume index; RVEDVI right ventricular end-diastolic volume index.

**Table 2:** Baseline characteristics in subjects without known comorbidities stratified by age.

**Variables**	**Control** **(n = 65)**	**18–40 years** **(n = 34)**	**41–60 years** **(n = 20)**	**> 60** **years (n = 11)**	**p-value**
	**Mean ± SD** **(95%-CI)**	**Mean ± SD** **(95%-CI)**	**Mean ± SD** **(95%-CI)**	**Mean ± SD** **(95%-CI)**	
***Clinical parameters***					
Age, years	42 ± 17	28 ± 6	51 ± 5	69 ± 7	**< .001**
Sex, female	24 (37%)	13 (20%)	6 (9%)	5 (8%)	0.68
BMI, kg/m^2^	24 ± 3	24 ± 4	24 ± 2	24 ± 3	0.75
***Laboratory markers***					
Creatinine, mg/dL	0.8 ± 0.2	0.8 ± 0.2	0.8 ± 0.2	0.7 ± 0.1	0.91
eGFR, ml/min/1.73m^2^	114 ± 25	121 ± 30	110 ± 21	99 ± 9	**0.03**
Hemoglobin, g/dL	14 ± 2	14 ± 2	14 ± 2	14 ± 2	0.46
Hematocrit, %	41 ± 4	42 ± 4	41 ± 4	41 ± 4	0.88
***Cardiac magnetic resonance imaging parameters***					
LV-EF, %	61 ± 9	61 ± 8	62 ± 9	59 ± 13	0.74
RV-EF, %	55 ± 9	55 ± 8	56 ± 10	53 ± 5	0.74
IVS, mm	10 ± 4	10 ± 4	10 ± 2	12 ± 5	0.14
LVEDVi, ml/m^2^	84 ± 18	85 ± 16	86 ± 21	75 ± 19	0.27
RVEDVI, ml/m2	87 ± 22	89 ± 22	87 ± 26	82 ± 13	0.70
Myocardial T1 times, ms	1006 ± 45	1005 ± 41	1019 ± 43	988 ± 59	0.20
Renal T1times, global, ms	1054 ± 53 (1040–1067)	1045 ± 61 (1024–1066)	1061 ± 43 (1042–1081)	1068 ± 37 (1043–1093)	0.34
Renal T1 times, upper, ms	1069 ± 58 (1054–1083)	1065 ± 58 (1045–1085)	1061 ± 62 (1032–1090)	1094 ± 51 (1060–1128)	0.28
Renal T1 times, middle, ms	1061 ± 55 (1047–1074)	1052 ± 48 (1035–1068)	1072 ± 60 (1044–1100)	1071 ± 68 (1025–1117)	0.36
Renal T1 times, lower, ms	1044 ± 58 (1030–1058)	1045 ± 61 (1022–1067)	1047 ± 52 (1023–1071)	1040 ± 57 (1001–1078)	0.95

**Abbreviations.** BMI, body mass index; NT-proBNP, N-terminal pro-brain natriuretic peptide; eGFR, estimated glomerular filtration rate; LV-EF, left ventricular ejection fraction; RV-EF, right ventricular ejection fraction; IVS, intraventricular septum; LVEDVi left ventricular end-diastolic volume index, RVEDVI right ventricular end-diastolic volume index.

In the control cohort, the mean global renal cortical T1 times were 1054 ± 53 ms (95% CI 1040–1067).

Renal cortical T1 times were consistent across the upper, middle, and lower cortical regions, with no significant segmental differences.

The average renal cortical T1 times were higher in women than in men (1068 ± 34 vs. 1046 ± 60 ms), but this difference was not statistically significant (p = 0.21). No significant differences were obsereved in renal cortical T1 times across age groups (p = 0.34).

No significant correlations were observed between renal cortical T1 times and hemoglobin (r = –0.10, p = 0.45) or hematocrit (r = –0.08, p = 0.54).

The intraclass correlation coefficient (ICC, two-way random, absolute agreement) was 0.81, indicating fair agreement between measurements.

## 4 Discussion

This study provides exploratory real-world reference estimates for renal cortical T1 times at 1.5 T in a cardiovascular population with preserved renal function and without major comorbidities.

Although women exhibited numerically higher renal cortical T1 times than men, this difference was not statistically significant. Similarly, no significant differences in T1 times were observed across age groups. These findings suggest that renal cortical T1 times are relatively stable across demographic subgroups in individuals with preserved renal function.

Our findings are in line with recent studies reporting relatively stable renal cortical T1 times across demographic subgroups in individuals without advanced renal disease [14]. Importantly, the present reference estimates were derived from patients representative of those encountered in daily clinical settings, thereby enhancing external validity. Integrating renal T1 mapping into standard CMR protocols may support earlier identification of patients at risk and support more individualized management strategies.

The present study reflects a real-world clinical cohort and provides an initial characterization of renal cortical T1 times. Future research should validate these findings in larger, multicentre cohorts with histopathologic correlation to further elucidate the underlying tissue mechanisms.

### 4.1 Application of T1 mapping

Initial applications of multiparametric imaging using mapping techniques such as T1 and T2(*) mapping have primarily focused on cardiac tissue. However, these approaches can also be applied to other organs, including the liver, skeletal muscle, and kidneys [3], [4], [5], [6], [7], [8], [15], [16]. The ability to non-invasively characterize tissue composition may ultimately enhance the understanding of shared disease pathways and enable more comprehensive risk stratification. Renal fibrosis is associated with adverse clinical outcomes. Native T1 times reflect a combination of tissue properties, including fibrosis, edema, and perfusion-related factors, which limit their specificity for individual pathological processes without histopathologic validation [2] [5] [17].

Several studies have reported correlations between renal T1 times and renal function parameters such as eGFR and serum creatinine, with promising preliminary results. In earlier studies, T1 times have been linked to structural changes, like fibrosis. However, their exact biological meaning is still unclear [3], [4], [5], [6], [7], [8]. To date, only one study has used renal biopsies to confirm the correlation between elevated renal T1 times and fibrosis [5]. Native T1 mapping is sensitive to multiple tissue properties, including fibrosis, edema, and amyloid deposition and should therefore not be interpreted as a direct marker of fibrosis without histopathologic validation [2].

To our knowledge, only one previous study has reported reference ranges for renal cortical T1 times [14]. The reference ranges established in our 1.5 T study differ from those reported in the prior 3.0 T study, which demonstrated higher renal cortical T1 times. This discrepancy likely reflects the influence of magnetic field strength, as myocardial T1 times have also been shown to be approximately 100 ms longer at 3.0 T compared to 1.5 T [18], [19]. Our results also demonstrated slightly higher renal cortical T1 times in females compared to males, although statistically not significant. These findings are consistent with previous studies investigating renal and myocardial T1 times [14] [20], [21], [22]. Earlier research has shown that differences in hematocrit are linked to changes in T1 times [23]. However, no significant associations were observed in the present cohort.

### 4.2 Technical considerations of T1 mapping

Parametric mapping techniques, including T1 and T2 mapping, enable voxel-wise quantitative assessment of tissue characteristics and are increasingly recognized as valuable diagnostic and prognostic imaging biomarkers [12] [24]. Two principal pathological mechanisms are associated with elevated T1 times: edema, as seen in acute infarction or inflammation, and expansion of the interstitial space due to fibrosis, scarring, or amyloid deposition [13], [24].

Without histopathologic validation or complementary perfusion-weighted imaging, attributing these changes solely to fibrotic remodelling remains speculative. Prospective studies combining native T1-weighted imaging with perfusion assessment and renal biopsy is warranted to differentiate these mechanisms.

The present findings may have translational relevance. Given the high prevalence of cardio-renal interactions, incorporating renal T1 mapping into standard CMR protocols could provide additional subclinical risk markers without extending scan time. Nevertheless, several technical and methodological issues must be addressed before clinical implementation, including vendor harmonization, and standardized ROI placement. Furthermore, the single-kidney approach employed here, although improving reproducibility, may not fully capture bilateral renal physiology.

Reproducibility analysis demonstrated fair agreement between measurements. In this study ROI size was adapted to the individual cortical thickness to ensure accurate placement within the cortex while minimizing partial volume effects. Due to anatomical variability and the use of cardiac short-axis images, ROI size was not strictly standardized.

## 5 Limitations

Our study establishes renal cortical T1 times real-world reference estimates at 1.5 T in a cardiovascular population without manifest kidney disease.

This study has several limitations. First, its retrospective, single-centre design may limit statistical power and generalizability. The reported real-world reference estimates are specific to the 1.5 T field strength and scanner model used and may not be directly applicable to other systems without calibration.

Second, although only subjects without known renal disease and with eGFR values ≥ 90 ml/min/1.73 m² were included, renal biopsies were not performed to exclude subtle fibrotic changes. Third, only the left kidney was analysed, as it is routinely and consistently visualized in the standard short-axis cardiac T1-mapping protocol. While this approach ensured uniform image quality and high reproducibility, it may not fully represent bilateral renal physiology.

Renal cortical T1 measurements may be affected by partial volume effects, particularly due to the thin cortical layer and the use of cardiac short-axis imaging planes. Despite careful ROI placement, inclusion of adjacent medullary tissue or perirenal fat cannot be entirely excluded, which may have influenced the measured T1 times. Medullary T1 times and corticomedullary differentiation were not assessed due to limited reproducibility on non-dedicated imaging planes.

Renal T1 times were derived from a MOLLI sequence optimized for cardiac imaging. No heart-rate correction or phantom validation was performed for renal measurements. Therefore, absolute renal T1 times may be influenced by sequence-specific factors.

Hydration status and diuretic use were not systematically recorded due to the retrospective study design and may have influenced renal T1 measurements.

Finally, the study population was not composed of healthy volunteers but of patients referred for CMR due to suspected cardiac disease. Although this may reduce generalizability, it also enhances clinical applicability, as the cohort reflects real-world cardiovascular populations. The use of a standardized imaging protocol and the rigorous exclusion of manifest renal disease strengthen internal validity, while future multicentre prospective studies are needed to confirm these findings.

## 6 Conclusion

This study provides real-world reference estimates for renal cortical T1 times at 1.5 T in a cardiovascular cohort with preserved renal function.

Opportunistic renal T1 assessment from routine CMR may provide additional, non-invasive information on renal tissue characteristics without extending scan time.

## Author contribution

The following individuals have contributed to the publication:

L.L. contributed to the study conception and design, data acquisition, image analysis, statistical analysis, interpretation of the data, and drafted the manuscript.

M.K. contributed to data acquisition and critically revised the manuscript.

K.C., D.C., N.C., K.M., P.M., S.L., G.A., B.D., L.C., and H.C. critically revised the manuscript.

K.A. contributed to the study conception, supervision, critical revision of the manuscript, and final approval.

All authors read and approved the final manuscript.

## Ethical Statement

This retrospective analysis was conducted within the framework of a prospective registry at the Medical University of Vienna, Department of Internal Medicine II, Division of Cardiology. The study was approved by the institutional ethics committee (EK#2036/2015) and was performed in accordance with the Declaration of Helsinki. Written informed consent was obtained from all participants prior to inclusion.

## Funding

This research did not receive any specific grant from funding agencies in the public, commercial, or not-for-profit sectors.

## CRediT authorship contribution statement

**L. Lunzer:** Writing – original draft, Methodology, Investigation, Formal analysis, Data curation, Conceptualization. **K. Mascherbauer:** Writing – review & editing, Data curation. **C. Kronberger:** Writing – review & editing. **C. Dona:** Writing – review & editing. **C. Nitsche:** Writing – review & editing. **M. Koschutnik:** Writing – review & editing. **M. Poledniczek:** Writing – review & editing. **L. Schmid:** Writing – review & editing. **A. Geppert:** Writing – review & editing. **D. Beitzke:** Writing – review & editing. **C. Loewe:** Writing – review & editing. **C. Hengstenberg:** Writing – review & editing. **A. Kammerlander:** Writing – review & editing, Supervision.

## Declaration of Competing Interest

The authors declare they have no conflict of interest
